# Ultra-Processed Foods and Drinks Consumption Is Associated with Psychosocial Functioning in Adolescents

**DOI:** 10.3390/nu14224831

**Published:** 2022-11-15

**Authors:** Marta Reales-Moreno, Pietro Tonini, Rosa M. Escorihuela, Montserrat Solanas, Sílvia Fernández-Barrés, Dora Romaguera, Oren Contreras-Rodríguez

**Affiliations:** 1 Medical Imaging, Girona Biomedical Research Institute (IdIBGi), Parc Hospitalari Martí i Julià-Edifici M2, 17190 Girona, Spain; 2 Department of Medical Sciences, School of Medicine, University of Girona, 17071 Girona, Spain; 3 Sostenipra Research Group (2017 SGR 1683), Unitat d’Excel·lència Maria de Maetzu MDM, CEX2019-000940-M, Institut de Ciència i Tecnologia Ambientals (ICTA-UAB), Universitat Autònoma de Barcelona, 08193 Bellaterra, Spain; 4 Department of Psychiatry and Legal Medicine, Faculty of Medicine, Universitat Autònoma de Barcelona, 08193 Bellaterra, Spain; 5 Institut de Neurociències, Universitat Autònoma de Barcelona, 08193 Bellaterra, Spain; 6 Physiology Unit, Department of Cell Biology, Physiology and Immunology, Faculty of Medicine, Universitat Autònoma de Barcelona, 08193 Bellaterra, Spain; 7 Agència de Salut Pública de Barcelona, 08193 Bellaterra, Spain; 8 Institut d’Investigació Sanitaria Illes Balears (IdISBa), 07120 Palma, Spain; 9 CIBER Fisiopatología de la Obesidad y Nutrición (CIBEROBN), 28029 Madrid, Spain; 10 Health Institute Carlos III (ISCIII) and CIBERSAM G17, 28029 Madrid, Spain

**Keywords:** ultra-processed foods and drinks, fruits and vegetables, physical activity, psychosocial functioning, adolescence

## Abstract

Adolescents show one of the highest rates of ultra-processed foods and drinks (UPF) consumption, and studies indicate an association between their consumption and internalizing problems. We aim to investigate whether UPF consumption associates with dysfunctions in other psychosocial domains, as well as sex effects. In 560 Spanish adolescents (14–17 years old), we assessed the UPF products consumed in the previous day, fruits and vegetables consumption (servings/day), and physical activity (days/week). Psychosocial functioning (total and subscales) was assessed through the Pediatric Symptom Checklist–Youth self-report. Associations between UPF and psychosocial functioning were assessed using linear regression models, adjusting for sex, age, fruits and vegetables consumption, and physical activity. Sex-specific associations were also explored. Participants reported a daily consumption of 7.72 UPF servings per day, with male adolescents showing higher consumption than female adolescents. Consumption of fruits and vegetables and physical activity levels were lower than recommended. Psychosocial impairment was present in 26.2% of the participants. Higher UPF consumption was associated with higher presence of depressive symptoms and internalizing and externalizing problems in the whole sample and in male adolescents. The present study supports previous studies suggesting that UPF consumption may interact with mental health problems and indicates that these effects may go beyond internalizing problems.

## 1. Introduction

The World Health Organization has warned that the consequences of not addressing mental health conditions during adolescence extend into adulthood [1]. Current epidemiological studies estimate that 1 in 7 (14%) adolescents experience mental health conditions, with depression, anxiety, and behavioral disorders being among the leading causes of illness and disability [1]. Therefore, identifying factors interacting with mental health problems is a high priority in developing preventive strategies in adolescents [2]. Poor diet quality, characterized by a high consumption of ultra-processed foods and drinks (UPF in brief), is a potentially modifiable risk factor for mental disorders [3].

UPF are ingredient formulations that result from series of industrial processes [4]. They contain no or relatively small amounts of minimally processed foods that conserve their nutritional properties. In general, most UPF have low nutrient densities and are poor sources of protein, dietary fiber, and micronutrients. At the same time, they have high energy densities, together with high contents of saturated and trans fatty acids, added sugars, and salt compared to unprocessed foods [5]. Moreover, UPF usually contain additives (i.e., sweeteners, colorants, emulsifiers) intended to intensify their sensory qualities and imitate the appearance of minimally processed foods—making them edible, palatable, highly attractive, and habit-forming [6]. Recent research has warned about the presence in UPF of chemicals acquired through contact materials, such as sophisticated packaging (e.g., bisphenol), and neo-formed contaminants generated during food processing practices [7].

Adolescents show one of the highest rates of UPF consumption, with recent studies estimating the percentage of their total energy intake obtained from UPF consumption being between 29% and 68% [8,9]. In Spain, the percentage of ultra-processed foods among all food purchases almost tripled between 1990 and 2010 (from 11.0% to 31.7%) [10]. In adolescents, UPF consumption is associated with specific nutritional deficiencies, including lower levels of micronutrients and vitamins [11], proteins, and dietary fiber, and higher ingestion of saturated and trans fatty acids, added sugars, and sodium [9,12]. Alongside these concerns, recent studies have started to shed light on the adverse effects that UPF consumption has on mental health [3,13]. Studies showed an association between UPF consumption and internalizing problems [14], depressive symptoms [15], anxiety-induced disturbances [16], and the risk for common mental health disorders [17]. Some of these effects have been reported to be sex-specific [14]. These findings are congruent with pre-clinical evidence that UPF components (i.e., nanosized particles contained in some additives, trans fatty acids, chemicals) impair several brain regions, including the hippocampus and the cortex, implicated in emotional processes [18].

In contrast, substantial evidence points toward the mental health benefits associated with healthy habits, including achieving the recommendations of fruits and vegetables consumption (≥5 servings/day, [19]) and physical activity [20]. For instance, a systematic review showed a relationship between healthy dietary habits and internalizing disorders in adolescents [21]. In another study, a low consumption of fruits and vegetables was associated with low mental health across adolescence [22]. Indeed, randomized controlled trials have demonstrated that healthy dietary changes can improve depression [23]. Physical activity is also identified as a key factor in reducing adverse physical, mental, and social health outcomes in the population [24]. In adolescents, more frequent physical activity and participation in sports independently contributed to greater well-being and lower levels of anxiety and depressive symptoms in both sexes [25].

Therefore, there is some preliminary evidence that UPF consumption may be related to poorer mental health, particularly to internalizing problems in adolescents [3]. However, there is a need to study whether UPF consumption may associate with dysfunctions in other psychosocial domains and whether its effects are different between sexes. The main objective of the present study was to provide estimations of the daily consumption of UPF and fruits and vegetables, as well as the weekly physical activity, in a large sample of Spanish adolescents. Then, we aimed to comprehensively explore the associations between UPF consumption and different domains of psychosocial functioning while adjusting for sex, age, fruits and vegetables consumption, and physical activity. Specific sex effects on the above associations were explored.

## 2. Methods and Materials

### 2.1. Study Overview and Participants

Five hundred and sixty adolescents from five high schools and educational centers participated in this cross-sectional study as part of the health education program of the involved centers. The study was conducted between February and April 2022 in Spain (in the region of Catalonia). Participants attended a brief presentation to familiarize them with the questionnaires. We also introduced the concept of servings, the types of UPF products, and the way they should provide the answers. Afterward, they completed three self-report questionnaires. These questionnaires compiled information regarding age, sex, UPF and fruits and vegetables consumption, physical activity, and psychosocial functioning. The biological sex variable used in this study was labeled as female, male, and indeterminate sex [26]. Indeterminate sex refers to unknown biological sex and included those who did not want to answer this question, those whose biology did not allow them to define themselves (e.g., intersex, medical syndromes, etc.), or any other personal case. Data were collected on a single day from Tuesday to Friday, to estimate the usual weekday diet and avoid dietary changes between weekdays and weekends [27]. No exclusion criteria were considered, so that the sample represents the population as much as possible. The information of the study participants is shown in Table 1. 

The Research Ethics Committee of Bellvitge University Hospital (Barcelona, Spain) approved this study protocol. All procedures were in accordance with the ethical standards established by the Declaration of Helsinki (Fortaleza, 2013). All participants were informed about the study’s purpose and gave their consent. The processing of personal data complies with current Spanish and European legal regulations on data protection.

### 2.2. UPF, Fruits and Vegetables Consumption, and Physical Activity

Participants received descriptive information and visual examples to help them identify and appropriately report the type of UPF products consumed. We used a screening tool previously designed for the specific assessment of the consumption of these products during the previous 24 h [28]. The original screening tool was slightly modified to reflect the types of UPF products available to the targeted Spanish adolescent population. This resulted in a list of 24 UPF products, which are listed in Table 2. Participants were asked to report if they had consumed each type of UPF products during the previous day. For each individual, we calculated the total UPF products consumed during the last 24 h, which ranged from 0 to 24.

Fruits and vegetables consumption was assessed as the frequency (i.e., number of days a week, from 0 to 7 days), and the quantity (i.e., number of standard servings in the days of consumption, from ‘almost never’ to ‘five or more servings’). Particularly, we asked the following questions: ‘In a regular week, on how many days do you eat fruits?’, and ‘How many servings of fruits did you eat in one day when you consumed them?’ The same questions were applied to the consumption of vegetables. From these questions, we derived the habitual servings per day of fruits and vegetables. The definitions of the fruits and vegetable groups were taken from the Food and Agriculture Organization of the United Nations (FAO) guidelines [29]. According to the number and size of the products, a standard serving has been identified using the graphical image taken by the database of the Spanish Food Safety and Nutrition Agency (AESAN) [30].

Physical activity was assessed as the number of practice days during a week (range 0 to 7 days). Particularly, we asked the adolescents, ‘How many days a week do you do physical activity?’ Since physical education is mandatory during secondary school, it was explicitly excluded from the quantification.

### 2.3. Psychosocial Functioning

Psychosocial functioning was assessed using the validated Spanish version of the Pediatric Symptom Checklist–Youth self-report (Y-PSC), for which the sensitivity is 95% and the specificity is 68% [31]. The Y-PSC has 35 items and uses a 3-point response format scale (‘Never’ = 0, ‘Sometimes’ = 1, or ‘Often’ = 2). Total and subscales scores (i.e., attention deficit and hyperactivity symptoms, depressive symptoms, behavior symptoms, anxiety symptoms, internalizing problems, externalizing problems, and attention problems) were derived from the Y-PSC [32]. A total score of 30 or above suggests a psychosocial impairment in the adolescent. The Internalizing subscale has a clinical cutoff of ≥5. The Externalizing and Attention subscales have a clinical cutoff of ≥7 [31]. If 1 to 3 items were left blank, each was scored 0. Five participants were excluded because 4 or more items were left blank, based on the Y-PSC scoring guidelines.

### 2.4. Statistical Analyses

All analyses were performed using IBM SPSS Statistics, version 23. The presence of outlier values in all the study variables was explored, and values > 2 standard deviation (SD) were eliminated from subsequent analyses. Data were used as numerical variables, and the descriptive analysis was explored with means, min-max values, and SD, in the total sample and by sex. We also characterized the proportion of participants that reported the consumption of each of the 24 UPF products. Differences in the study variables between sexes (female, male, indeterminate) were tested with an analysis of variance (ANOVA) test, followed by independent sample t-tests to evaluate differences among groups. The association between UPF consumption, physical activity, and the consumption of fruits and vegetables was assessed through Pearson correlation analyses. Also, we categorized the participants’ UPF consumption in the previous day into quartiles to represent low (0–4), low-medium (5–6), high-medium (7–8), and high consumption (9–24). We compared the level of physical activity and the consumption of fruits and vegetables between those participants that fall in the low vs. the high quartiles using independent samples t-tests.

Linear regression analyses were used to assess the association between UPF consumption (total consumption) and psychosocial functioning (total and subscales Y-PSC scores). Model 1 included as covariates sex and age. Model 2 was further adjusted for physical activity and fruits and vegetables consumption. All models were repeated dividing by sex.

## 3. Results

The demographic, dietary, and physical activity information of the study participants, as well as for the sample stratified by sex, are provided in Table 1. Participants were between 14 and 17 years old, most of them female adolescents (58.4% female adolescents, 38.8% male adolescents, 9% indeterminate).

### 3.1. UPF Consumption

**Figure 1:**
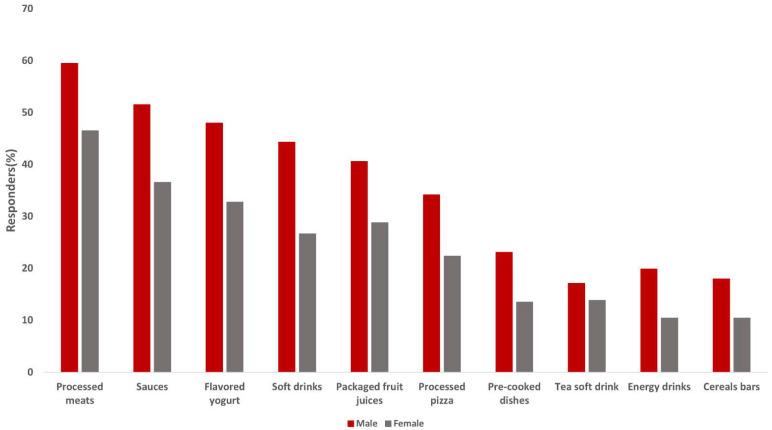
Graphical display of the percentage of male and female adolescents consuming those ultra-processed foods and drinks that statistically differ between these groups.

Participants reported the consumption of 7.72 UPF in the previous 24 h. Scores ranged from 0 to 24 categories, but 3 (8.6%), 4 (10%), 5 (12%), and 6 (12.3%) were the most common. Total UPF consumption was higher in male than in female adolescents (Table 1). Table 2 describes the proportion of participants consuming each of the UPF products on the day prior to the interview. Over 60% of the participants consumed cold meats and similar. From 50–60% reported the consumption of cookies and processed meats, 40–50% chocolate products, drinks, snacks, and sauces, and 30–40% consumed flavored yogurts, processed breads, bakery products, sugary breakfast cereals, soft drinks, packaged fruit juices, and processed fries. Lower percentages of consumption (<15%) were observed for energy drinks, margarine, cereal bars, and alcoholic drinks. Between sexes, male adolescents consumed significantly more sauces, flavored yogurts, soft and energy drinks, packaged fruit juices, processed pizza and meats, and cereal bars than female adolescents (Figure 1, Table 2). Those in the indeterminate group consumed significantly more tea soft drinks than male and female adolescents, and more energy drinks than female adolescents. On the contrary, female adolescents do not have a significantly higher consumption of any UPF products compared with the male adolescents and the indeterminate group.

### 3.2. Fruits and Vegetables Consumption, and Physical Activity

**Figure 2:**
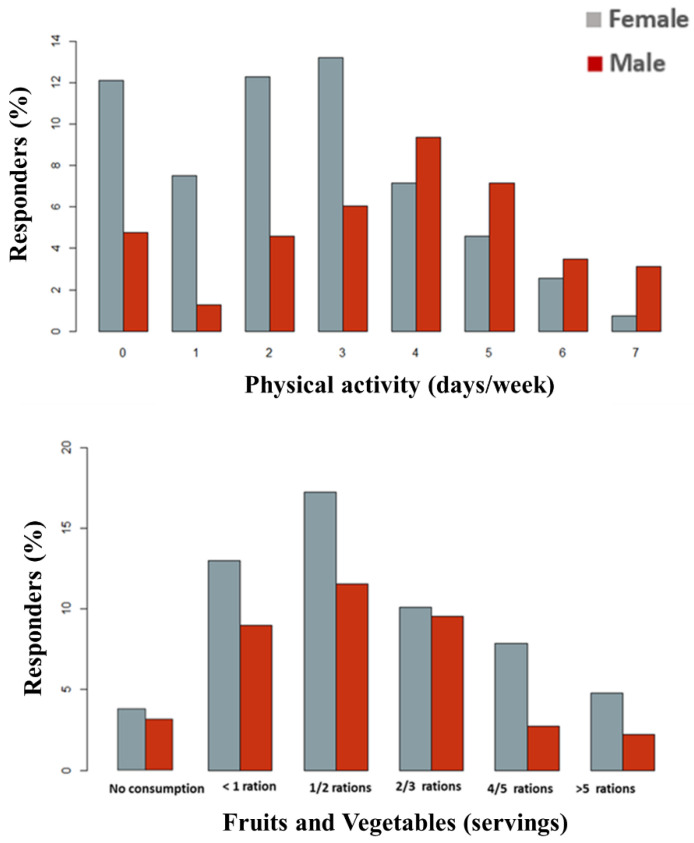
Physical exercise (days/week) and consumption of fruits and vegetables (servings/day) among the study participants stratified by sex.

Participants reported their consumption of fruits and vegetables (1.93 servings/day) and amount of physical activity (2.9 days/week) (Figure 2; Table 1). Male adolescents reported significantly more physical activity than the female adolescents, while this latest group reported a significantly higher consumption of fruits and vegetables compared to the male adolescents. There was a positive correlation between level physical activity and fruit and vegetable consumption (r = 0.177, *p* = 0.000), but no association emerged between these variables and the consumption of UPF. A total of 163 subjects fall in the low quartile of consumption, 152 in the low-medium, 125 in the high-medium, and 120 in the high. Participants in the high quartile of UPF consumption showed lower consumption of fruits and vegetables compared to those in the lowest quartile (1.25 vs. 2.24 servings/day, respectively, *p* = 0.027), but no differences in the level of physical activity (*p* > 0.05).

### 3.3. Psychosocial Functioning

We found psychosocial impairment in 26.2% of the participants. Across specific subscales, 33.9% showed internalizing problems, 3.9% externalizing problems, and 9.5% attention problems (Table 1). Female compared to male adolescents showed a higher presence of total psychosocial impairment and scored higher for all symptoms and problems, except for externalizing problems (Table 1). Those in the indeterminate group showed a higher presence of behavioral symptoms and externalizing problems than the female adolescents, and higher presence of total psychosocial impairment, attention deficit and hyperactivity symptoms, depressive and behavioral symptoms, and externalizing problems than the male adolescents.

### 3.4. Association between UPF Consumption and Psychosocial Functioning

**Figure 3:**
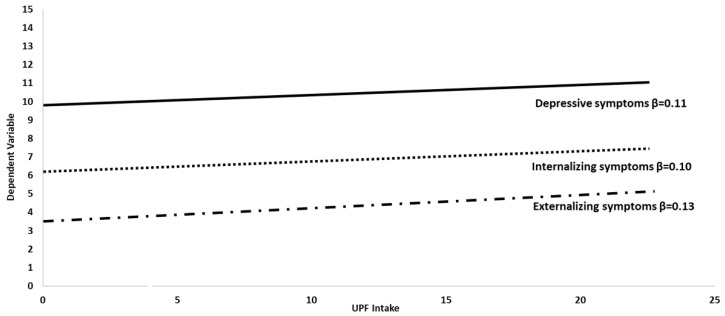
Association between the total consumption of ultra-processed foods and drinks and depressive symptoms, internalizing and externalizing problems in all participants, adjusting for age, sex, fruits and vegetables consumption, and physical activity (Model 2). ß are standardized coefficients adjusted for sex, age, physical activity, and fruits and vegetables intake as in Model 2.

Total UPF consumption showed a trend towards a positive association with the total score of the Y-PSC, and a significant positive association with depressive symptoms and internalizing and externalizing problems (Figure 3, Table 3). In the sex-stratified models, these associations remained significant only in male adolescents. The indeterminate sex showed a positive association between UPF consumption and attention deficit and hyperactivity symptoms, behavior symptoms, and externalizing problems. These associations were also significant when adjusting for sex, age, physical activity, and fruits and vegetables consumption, as appropriate.

## 4. Discussion

In this cross-sectional study, Spanish adolescents from the general population reported a daily consumption of 7.72 UPF servings per day, with male adolescents showing higher consumption than female adolescents. More than 50% of the participants reported the consumption of cold and processed meats and cookies. Consumption of fruits and vegetables and physical activity levels were overall lower than recommended, although female adolescents reported the highest consumption of fruits and vegetables, and male adolescents reported more physical activity per week. Psychosocial impairment was present in 26.2% of the participants, predominantly of depressive symptoms. Female adolescents overall reported more psychosocial impairment, specifically concerning depressive symptoms, anxiety symptoms and internalizing problems congruently with previous research, including that performed with a Spanish sample aged 15–24 years [33,34]. Higher UPF consumption was associated with a higher presence of depressive symptoms and internalizing and externalizing problems in the whole sample. In sex-stratified analyses, the associations were significant only in male adolescents. Overall, associations remained significant when adjusting for sex, age, fruits and vegetables consumption, and physical activity.

The consumption of UPF products on the day prior to the interview is higher than that reported by a previous study using the same assessment instrument in a sample of Brazilian adults [28]. In particular, the adolescents in our study showed a higher range of UPF consumption, with most reporting the consumption of five or six of these products, whereas dos Santos Costa and colleagues [28] reported a common consumption of between one and three products. The higher consumption found in male adolescents herein is congruent with a previous study with Canadian participants including different age groups [35], although female Spanish adults have been reported to have a higher UPF consumption [36]. Most products consumed by adolescents were cold meats, cookies, processed meats, chocolate products, different types of snacks, chocolate drinks, and sauces. It is interesting that, among adults, the study of Romero-Ferreiro also found processed meats and cookies to be main groups of UPF consumed in Spain [36]. In agreement, Brazilian participants in the study of dos Santos Costa [28] and Belgian children [37] also showed these foods among their most consumed. Differences in the population of study (e.g., age, sex), cultural factors, and the country food system may explain differences in the amounts of UPF consumption and types of products across the studies.

The association found between UPF consumption, internalizing problems, and depression symptoms among adolescents herein is congruent with previous studies conducted in similar samples of Brazilian adolescents [14,15,16]. These studies obtained similar results, although they used different questionnaires for the assessment of UPF consumption (a food frequency questionnaire in [14], and self-reported questionnaires of consumption in the previous seven days [15,16]). In addition, we found an association between UPF consumption and externalizing problems, which include behaviors suggestive of conduct disorder or oppositional defiant disorder, including fighting or teasing others, not listening to rules, refusing to share, not understanding others’ feelings, and taking things that belong to others. This is a new finding not reported in previous studies using a food classification based on the level of food processing. However, this finding may be congruent with previous research showing an association between the consumption of low nutritional content (i.e., junk foods) and violent behaviors in adolescent samples [38,39]. Further research is needed to determine the specific effects of food processing beyond nutritional variables. No associations with attention-deficit/hyperactivity symptoms were found in our sample of adolescents. This finding is contrary to a previous cohort-based study in humans that found UPF consumption during pregnancy to be associated with increased attention-deficit/hyperactivity symptoms in the offspring at the age of eight [40]. Further evidence supporting the idea that eating ultra-processed foods, as part of an unhealthy eating pattern, may associate with attention-deficit/hyperactivity symptoms comes from a meta-analysis [41].

Associations with depression symptoms, internalizing and externalizing problems remained significant only in the group of male adolescents. Although the reason why this association was significant in the male but not in the female adolescents cannot be answered by the present study, some hypotheses can be raised. For instance, male adolescents reported higher consumption of some UPF products. These included sugar- and artificially-sweetened beverages (i.e., soft and energy drinks and packaged fruit juices) and processed meats that a previous meta-analysis [42] and a systematic review [43] have associated with the risk of depression or depressive symptoms (e.g., feeling worthless). Two of the studies considered included adolescents [38,44]. Depressive symptoms, as well as aggression, were also associated with a higher consumption of fast food in the study of Zahedi and colleagues [38], including pizza, which was more often consumed by the male adolescents herein. However, these studies did not explore for sex-specific effects. Differences in brain development between male and female adolescents, as well as the described dimorphic states of the brain should be considered to understand the specific effect of food processing [45,46]. For example, a recent study demonstrates a sexually divergent process of the adolescent development of a cortico-subcortical system that was relevant to depression [47]. In addition, differential sex-effects of food processing on potential biological mechanisms that have been associated with mental diseases should be also explored (e.g., monoamine synthesis, inflammation processes, hypothalamic-pituitary-adrenal axis regulation, and neurogenesis). The previous should be considered together with the fact that female adolescents showed a moderate consumption of UPF products compared to the other groups, but the highest consumption of fruits and vegetables. Conversely, although male adolescents showed more days of physical activity, the higher consumption of UPF products in this group was accompanied by a low consumption of fruits and vegetables.

Despite the evidence in the literature related to the protective effect of physical activity and healthy dietary patterns on mental health [21,23,24,25], the associations found with UPF consumption remained even when adjusting for these variables, in congruence with similar studies on adolescents [14]. However, it is important to note that the average consumption of fruits and vegetables was lower than that indicated by the international guidelines [19]. Indeed, the participants with the highest UPF consumption also showed lower consumption of fruits and vegetables, compared to those with the lowest UPF consumption, according to previous research [48]. However, and in contrast with previous studies [17,49], we did not find an association between UPF consumption and physical exercise. Only 10% of the participants in the present study met the established recommendations of 7 days of physical activity a week [20].

The advantages of this study include the detailed assessment of UPF products consumed in the 24 h before the assessment, as well as the large sample of adolescents assessed. The UPF products included were carefully defined based on a previously validated tool [28], which was revised and adapted to Spanish UPF products by a panel of nutritionists (SFB and DR). Therefore, the results are able to show the UPF consumption of a specific, targeted population. However, the assessment could be improved by also asking participants about the amount consumed within each of the UPF product categories, as it is uncertain whether the specific associations in male adolescents may be linked to higher amounts of UPF consumption. Similarly, further studies should improve the measurement of physical activity by coding its intensity and duration, and not only its frequency, which may facilitate comparison with previous studies using similar samples of adolescents [14,16]. Additional information about participants’ whole diets may also complement the results in the present study. Finally, the findings in the indeterminate sex, although reported, should be replicated in larger samples of participants. Other limitations refer to the lack of additional confounding variables that also associate with mental health, as, for instance, educational level, smoking, or the presence of prior stressful life events. Finally, the cross-sectional design of the present study cannot shed light on causality between UPF consumption and the variables explored. The present study should be complemented by additional research to understand the effect of prolonged UPF consumption on mental health.

## 5. Conclusions

The present cross-sectional study supports previous studies suggesting that UPF consumption may interact with mental health problems. Furthermore, associations in the present study suggest that the effects of food processing on mental health may extend beyond internalizing problems, also increasing the presence of externalizing problems. In addition, future studies in adolescents and with instruments assessing the amount of long-term UPF consumption are warranted to confirm our findings on mental health, as well as the sex-specific effects.

## Figures and Tables

**Table 1:** Demographic and questionnaires information of the study participants.

	Total (N = 560)	Male (N = 217)	Female (N = 327)	Indeterminate (N = 16)	*p*
Age (years) ^a^	15.24 ± 1.11	15.26 ± 1.13	15.25 ± 1.10	14.67 ± 0.98	0.129
UPF consumption (number products/24 h)	7.72 ± 5.10	8.67 ± 5.11	7.06 ± 4.97 ^+++^	8.31 ± 5.68	0.001
Physical activity (days/week)	2.90 ± 1.96	3.63 ± 1.99	2.39 ± 1.78 ^+++^	3.25 ± 1.91	<0.001
Fruits and vegetables consumption (servings/day)	1.93 ± 1.59	1.78 ± 1.45	2.06 ± 1.69 ^+^	1.24 ± 1.06	0.027
**Psychosocial functioning** ^b^					
Y-PSC total score	24.92 ± 9.06	22.28 ± 8.26 **	26.49 ± 8.87 ^+++^	28.69 ± 14.10	<0.001
Attention deficit and hyperactivity symptoms score	6.81 ± 3.11	6.44 ± 3.01 *	6.98 ± 3.09 ^+^	8.31 ± 4.27	0.022
Depressive symptoms score	9.41 ± 4.59	8.08 ± 4.10 *	10.21 ± 4.61 ^+++^	10.88 ± 6.27	<0.001
Behavior symptoms score	5.44 ± 2.92	5.06 ± 2.78 **	5.60 ± 2.87 ^+^	7.38 ± 4.66 ^^^	0.003
Anxiety symptoms score	6.36 ± 2.37	5.66 ± 2.21	6.84 ± 2.31 ^+++^	6.31 ± 3.40	<0.001
Internalizing problems score	4.50 ± 2.33	3.46 ±1.99	5.20 ± 2.24 ^+++^	4.44 ± 2.97	<0.001
Externalizing problems score	3.33 ± 2.16	3.29 ± 2.06 *	3.28 ± 2.13	4.75 ± 3.49 ^^^^	0.028
Attention problems score	5.14 ± 1.91	4.94 ± 1.87	5.30 ± 1.90 ^+^	4.75 ± 2.35	0.065

Mean ± standard deviations are provided. UPF, Ultra-processed foods and drinks; Y-PSC, Pediatric Symptom Checklist–Youth Report. ^a^ Age provided for N = 530/560 ^b^ Data from Y-PSC provided for N = 555/560. + *p* < 0.05, +++ *p* < 0.001 vs. male adolescents. * *p* < 0.05, ** *p* < 0.01 vs. indeterminate group. ^ *p* < 0.05, ^^ *p* < 0.01 vs. female adolescents.

**Table 2:** Percentage of participants consuming each of the 24 ultra-processed foods and drinks on the day prior to the interview, for all participants and stratifying by sex.

Product	Total (N = 560)	Male (N = 217)	Female (N = 327)	Indeterminate (N = 16)	*p*
Cold meats. Fuet, salami, mortadella, packaged sweet ham, pâté, and/or other similar.	353 (63.04%)	141 (64.98%)	203 (62.08%)	9 (56.25%)	0.673
Cookies. Plain cookies, cookies with chocolate.	302 (53.93%)	116 (53.46%)	180 (55.05%)	6 (37.50%)	0.384
Processed meats. Frankfurt sausages, hamburgers, fish or meat sticks, and/or other similar.	292 (52.14%)	129 (59.45%)	152 ^++^ (46.48%)	11 (68.75%)	0.005
Chocolate products. Chocolate, chocolate bars, and other chocolate products (including chocolate creams).	276 (49.29%)	110 (50.69%)	160 (48.93%)	6 (37.50%)	0.585
Snacks. Snacks, crackers, potato chips, and other salty or flavored snacks.	270 (48.21%)	103 (47.47%)	159 (48.62%)	8 (50.00%)	0.956
Chocolate drinks. Powdered chocolate drinks, or packaged with or without milk.	258 (46.07%)	101 (46.54%)	146 (44.65%)	11 (68.75%)	0.166
Sauces. Mayonnaise, ketchup, mustard, and/or other similar.	236 (42.14%)	111 (51.15%)	119 ^+++^ (36.39%)	6 (37.50%)	0.003
Flavored yogurts.	218 (38.93%)	104 (47.93%)	107 ^+++^ (32.72%)	7 (43.75%)	0.002
Processed breads. Sliced, hamburger, or hot dog bread.	206 (36.79%)	88 (40.55%)	113 (34.56%)	5 (31.25%)	0.328
Bakery. Bakery products (e.g., donuts, croissants, etc., except the homemade products).	206 (36.79%)	83 (38.25%)	119 (36.39%)	4 (25.00%)	0.556
Sugary breakfast cereals.	197 (35.18%)	83 (38.25%)	107 (32.72%)	7 (43.75%)	0.321
Soft drinks. Sugary drinks, light and/or without sugar. Carbonated drinks with or without flavors (e.g., cola, lemon, orange, etc.). Non-carbonated drinks (e.g., isotonic drinks with flavors, etc.).	188 (33.57%)	96 (44.24%)	87 ^+++^ (26.61%)	5 (31.25%)	<0.001
Packaged fruit juices. Fruit juices with or without dairy products, fruity drinks.	186 (33.21%)	88 (40.55%)	94 ^++^ (28.75%)	4 (25.00%)	0.013
Processed fries. Frozen fries or from fast-food chains.	171 (30.54%)	77 (35.48%)	89 (27.22%)	5 (31.25%)	0.122
Sweets. Gummies, lollipops, and other sweets.	160 (28.57%)	67 (30.88%)	87 (26.61%)	6 (37.50%)	0.406
Processed pizzas. Pre-frozen, frozen, or chain-restaurant pizza.	151 (26.96%)	74 (34.10%)	73 ^++^ (22.32%)	4 (25.00%)	0.010
Ice creams.	116 (20.71%)	53 (24.42%)	59 (18.04%)	4 (25.00%)	0.182
Pre-cooked dishes. Frozen pre-cooked dishes such as lasagna and/or other similar.	99 (17.68%)	50 (23.04%)	44 ^++^ (13.46%)	5 (31.25%)	0.006
Tea soft drinks.	88 (15.71%)	37 * (17.05%)	45 (13.76%)	6 ^^ (37.50%)	0.031
Instant soups. Noodles or other instant soups.	87 (15.54%)	40 (18.43%)	44 (13.46%)	3 (18.75%)	0.275
Energy drinks. Energy drinks, with stimulants, with or without sugar.	81 (14.46%)	43 (19.82%)	34 ^++^ (10.40%)	4 ^ (25.00%)	0.004
Margarine.	76 (13.57%)	33 (15.21%)	40 (12.23%)	3 (18.75%)	0.508
Cereal bars.	73 (13.04%)	39 (17.97%)	33 ^++^ (10.09%)	1 (6.25%)	0.020
Alcoholic drinks. Liquors, and/or other similar.	35 (6.25%)	16 (7.37%)	16 (4.89%)	3 (18.75%)	0.056

Percentages correspond to: ([total of participants consuming an UPF product/total number of participants] × 100). ++ *p* < 0.01, +++ *p* < 0.001 vs. male adolescents. * *p* < 0.05 vs. indeterminate group. ^ *p* < 0.05, ^^ *p* < 0.01 vs. female adolescents.

**Table 3:** Association between total consumption of ultra-processed foods and drinks and psychosocial functioning in all participants and stratifying by sex.

(N = 530)	MODEL 1 (Adjusted for Sex and Age)									MODEL 2 (Adjusted for Sex, Age, Physical Activity, and Fruits and Vegetables Intake)								
	ß			*p*			95% CI (Lower Bound, Upper Bound)			ß			*p*			95% CI (Lower Bound, Upper Bound)		
Y-PSC total	0.085			0.052			−0.001, 0.290			0.084			0.053			−0.002, 0.288		
Attention deficit and hyperactivity symptoms	0.051			0.239			−0.021, 0.082			0.050			0.248			−0.021, 0.082		
Depressive symptoms	0.107			0.013			0.019, 0.167			0.107			0.014			0.019, 0.166		
Behavior symptoms	0.076			0.079			−0.005, 0.090			0.074			0.087			−0.006, 0.088		
Anxiety symptoms	0.010			0.812			−0.034, 0.043			0.013			0.760			−0.032, 0.044		
Internalizing symptoms	0.101			0.020			0.007, 0.079			0.104			0.017			0.008, 0.080		
Externalizing symptoms	0.138			0.002			0.022, 0.092			0.135			0.002			0.021, 0.091		
Attentional symptoms	−0.042			0.340			−0.046, 0.016			−0.040			0.356			−0.046, 0.017		
**Stratifying for Sex**	**MODEL 1′** **(Adjusted for Age)**									**MODEL 2′** **(Adjusted for Age, Physical Activity, and Fruits and Vegetables Intake)**								
	**Male** **(N = 201)**			**Female** **(N = 314)**			**Indeterminate** **(N = 15)**			**Male** **(N = 201)**			**Female** **(N = 314)**			**Indeterminate** **(N = 15)**		
	**ß**	**P**	**95% CI**	**ß**	**P**	**95% CI**	**ß**	**P**	**95% CI**	**ß**	**P**	**95% CI**	**ß**	**P**	**95% CI**	**ß**	**P**	**95% CI**
Y-PSC total	0.122	0.085	−0.027, 0.416	0.036	0.523	−0.134, 0.263	0.453	0.090	−0.169, 2.076	0.124	0.079	−0.023, 0.421	0.028	0.615	−0.147, 0.249	0.491	0.063	−0.065, 2.159
Attention deficit and hyperactivity symptoms	−0.014	0.844	−0.090, 0.074	0.053	0.353	−0.036, 0.101	0.576	0.025	0.056, 0.691	−0.014	0.838	−0.091, 0.074	0.051	0.365	−0.037, 0.100	0.581	0.023	0.060, 0.694
Depressive symptoms	0.169	0.017	0.025, 0.245	0.056	0.324	−0.051, 0.154	0.403	0.136	−0.144, 0.939	0.172	0.015	0.027, 0.247	0.047	0.404	−0.059, 0.145	0.453	0.090	−0.081, 0.983
Behavior symptoms	0.111	0.117	−0.015, 0.131	0.004	0.949	−0.062, 0.066	0.599	0.018	0.089, 0.808	0.108	0.126	−0.015, 0.131	−0.005	0.934	−0.065, 0.060	0.620	0.014	0.115, 0.837
Anxiety symptoms	0.010	0.884	−0.055, 0.064	0.023	0.685	−0.041, 0.062	−0.030	0.916	−0.330, 0.298	0.014	0.844	−0.053, 0.067	0.020	0.728	−0.042, 0.061	−0.001	0.997	−0.310, 0.308
Internalizing symptoms	0.187	0.008	0.019, 0.125	0.074	0.191	−0.017, 0.083	0.062	0.827	−0.260, 0.320	0.190	0.007	0.023, 0.127	0.063	0.265	−0.022, 0.079	0.133	0.637	−0.219, 0.345
Externalizing symptoms	0.177	0.012	0.015, 0.121	0.068	0.231	−0.019, 0.077	0.569	0.027	0.044, 0.612	0.174	0.014	0.014, 0.120	0.062	0.274	−0.021, 0.073	0.585	0.022	0.058, 0.627
Attentional symptoms	−0.116	0.101	−0.090, 0.008	−0.003	0.959	−0.044, 0.041	0.175	0.534	−0.147, 0.270	−0.115	0.105	−0.089, 0.009	−0.003	0.952	−0.044, 0.041	0.186	0.508	−0.141, 0.271

Standardized ß coefficients and 95% CI estimated using linear regression models. Significant and trend *p*-values are shown in bold (*p* < 0.05) and italics, respectively. UPF, Ultra-processed foods and drinks; Y-PSC, Pediatric Symptom Checklist–Youth Report. Adjusting by age N = 530.

## Data Availability

Data is available under request.
